# Ultrafast Time Dynamics of Plasmonic Fractional Orbital Angular Momentum

**DOI:** 10.1021/acsphotonics.3c01036

**Published:** 2023-11-14

**Authors:** Thomas Bauer, Timothy J. Davis, Bettina Frank, Pascal Dreher, David Janoschka, Tim C. Meiler, Frank-J. Meyer zu Heringdorf, L. Kuipers, Harald Giessen

**Affiliations:** † Kavli Institute of Nanoscience Delft, Delft University of Technology, Delft 2628 CJ, The Netherlands; ‡ School of Physics, University of Melbourne, Parkville, Victoria 3010, Australia; § 4-th Physics Institute and Research Center SCoPE, University of Stuttgart, 70569 Stuttgart, Germany; ∥ Faculty of Physics and Center for Nanointegration, Duisburg-Essen (CENIDE), University of Duisburg-Essen, 47048 Duisburg, Germany

**Keywords:** plasmonic angular momentum, fractional orbital angular momentum, time-dynamics of 2D vortices, photoemission electron microscopy, near-field scanning optical microscopy

## Abstract

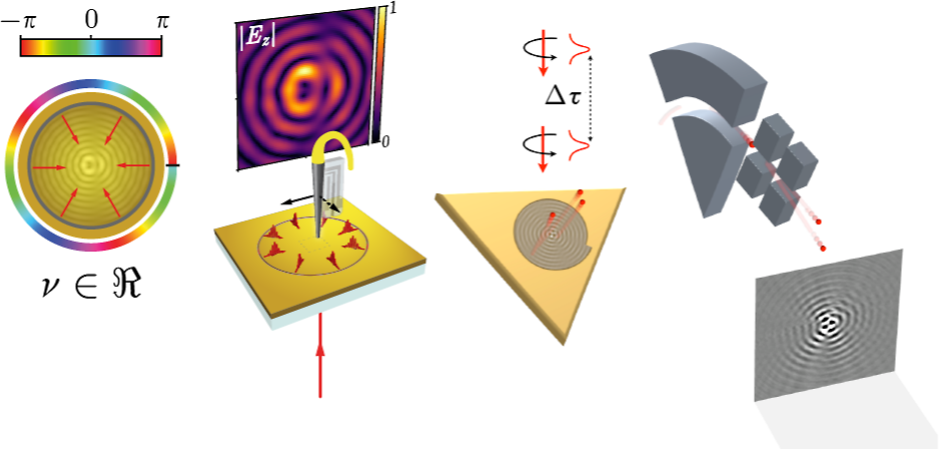

The creation and manipulation of optical vortices, both in free space and in two-dimensional systems such as surface plasmon polaritons (SPPs), has attracted widespread attention in nano-optics due to their robust topological structure. Coupled with strong spatial confinement in the case of SPPs, these plasmonic vortices and their underlying orbital angular momentum (OAM) have promise in novel light–matter interactions on the nanoscale with applications ranging from on-chip particle manipulation to tailored control of plasmonic quasiparticles. Until now, predominantly integer OAM values have been investigated. Here, we measure and analyze the time evolution of fractional OAM SPPs using time-resolved two-photon photoemission electron microscopy and near-field optical microscopy. We experimentally show the field’s complex rotational dynamics and observe the beating of integer OAM eigenmodes at fractional OAM excitations. With our ability to access the ultrafast time dynamics of the electric field, we can follow the buildup of the plasmonic fractional OAM during the interference of the converging surface plasmons. By adiabatically increasing the phase discontinuity at the excitation boundary, we track the total OAM, leading to plateaus around integer OAM values that arise from the interplay between intrinsic and extrinsic OAM.

## Introduction

The ability to create helical wavefronts^1,2^ confined to two-dimensional (2D) surfaces^3−5^ has recently gained widespread interest for accessing novel types of light–matter interaction on the nanoscale.^6−13^ While 2D surface waves containing optical vortices with integer orbital angular momentum (OAM) present as promising candidates for dedicated on-chip interfaces,^4,14,15^ enhanced sensors^16,17^ or sources for microparticle manipulation,^7,8,18^ removing the integer constraint on the OAM leads to an even richer set of dynamically changing field structures exhibiting fractional vortex modes. Although the general steady-state structure of these surface waves with fractional OAM and their composition from an infinite amount of integer OAM modes was recently shown numerically^19−22^ and experimentally,^23^ the intricate time dynamics of these fascinating surface modes has so far eluded any experimental investigation.

In this article, we experimentally visualize the dynamic time evolution of surface plasmon polariton (SPP) waves with non-integer OAM both in their steady-state distribution as well as during the buildup and decay resulting from ultrashort pulse excitation. By tracing the wave field in time via phase- and polarization-resolved near-field scanning optical microscopy (NSOM)^24−26^ as well as pump–probe based two-photon photoemission electron microscopy (2PPE–PEEM),^11,27−44^ we reveal intricate plasmonic vortex trajectories. We discern the differences between rotational symmetric eigenmodes for integer plasmonic OAM values and the time-dependent interference of several dominantly contributing eigenmodes for the fractional case. During the ultrafast buildup and decay of the plasmonic OAM, we observe an eigenmode content condensation as the fractional OAM content of the field reaches its maximum. This direct observation of the ultrafast time dynamics of fractional OAM SPPs gives detailed insight into the creation of non-integer OAM in lower-dimensional systems and the underlying dynamics of the plasmonic vortices and might allow for a versatile platform to study topologically analog concepts such as SPP realizations of the Aharonov–Bohm effect^31,32^ or fractional quantum Hall states^33^ as well as 2D chiral structures with broken time-reversal symmetry associated with fractional modal winding numbers.^34^

## Results and Discussion

### Time Dynamics of Plasmonic Vortices

Extracting ultrafast time dynamics of electromagnetic fields has a long history in free-space optics and has led to several breakthrough discoveries in solid-state physics.^35^ Experimentally retrieving such time information from the highly confined field distribution of bound surface waves with spatial features on the nanoscale requires simultaneous control over interferometric excitation geometries as well as access to the confined field distribution via near-field probes^25,26^ or nonlinear processes such as in 2PPE–PEEM.

**Figure 1:**
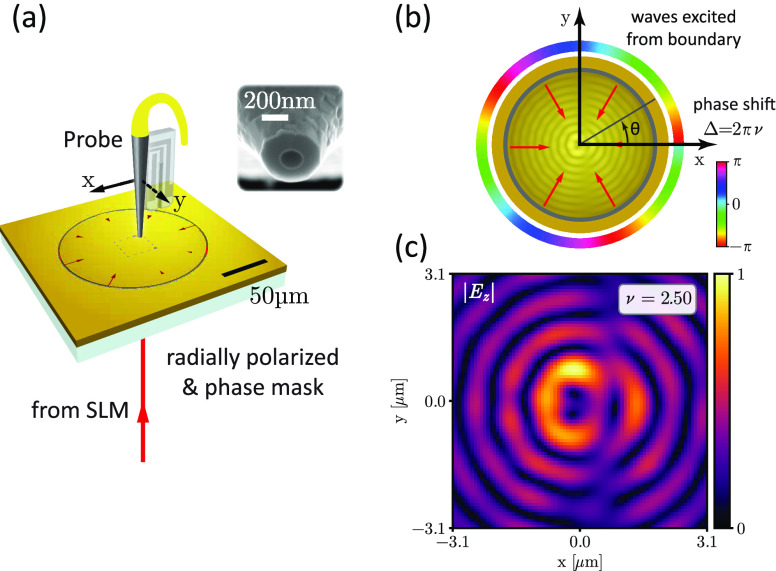
Experimental generation of plasmonic fractional OAM modes and observation of their steady-state time dynamics. (a) Sketch of the optical near-field setup. (b) Geometry of the excitation boundary and imprinted angular-dependent phase ramp. (c) Example amplitude distribution of the plasmonic wave function *A*_ν_(*r*,θ) ∝ *E*_*z*,ν_(*r*,θ) for an excitation phase step of ν = 2.5, experimentally extracted via NSOM.

To experimentally visualize the complex steady-state rotational dynamics of plasmonic fractional OAM, we perform aperture-based phase- and polarization-resolved near-field microscopy^24^ under continuous-wave illumination at an excitation wavelength λ of 1550 nm (see Figure 1a for a sketch of the experimental concept). An arbitrary excitation phase discontinuity Δ (see Figure 1b) is created by imprinting a geometric phase via a spatial light modulator onto the wave front of a radially polarized light beam (see the Supporting Information for details on the optical setup). This light beam illuminates a circular excitation boundary from below in an optically thick gold film. The boundary excitation launches SPP waves with an angular-dependent geometric phase delay ϕ(θ) = νθ, with θ the angle of the boundary position with respect to the *x*-axis of the coordinate system defined by the center of the boundary, which creates a converging SPP field with a fractional excitation step ν and a complex non-centrosymmetric spatial distribution for any non-integer value of ν. By raster scanning a near-field probe over the center of the generated SPP distribution and detecting the amplitude and phase of the local electromagnetic field, we measure the wave function of the plasmonic fractional OAM wave (see Figure 1c for an exemplarily retrieved amplitude distribution for ν = 2.5 and the Supporting Information for details about the experimental setup as well as further raster scans of the resulting plasmonic vortex fields). This phase-resolved detection enables us to visualize the steady-state time dynamics of the plasmonic fractional OAM wave.

**Figure 2:**
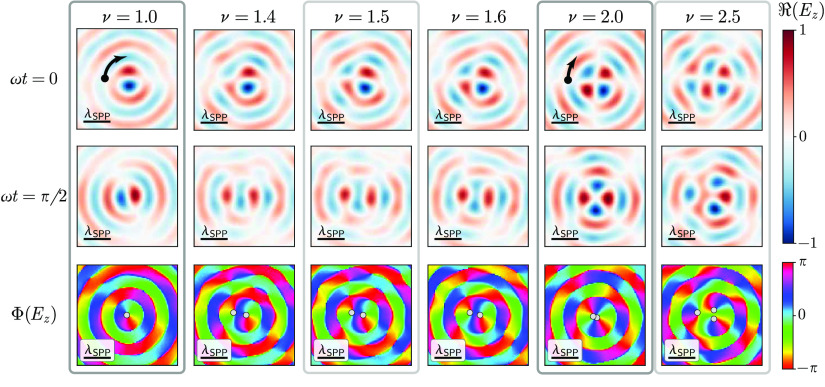
Experimentally measured steady-state time dynamics of integer and fractional OAM modes via phase- and polarization-resolved NSOM. The real part of the wave function *A*_ν_ ∝ *E*_*z*,ν_ is shown for time instants ω*t* = 0, π/2, and π within one optical cycle of the plasmon wave with angular frequency ω, for different excitation phase steps of ν = 1, 1.4, 1.5, 1.6, 2, and 2.5(see Visualization **1–6** for time evolution movies of one full optical cycle). For integer OAM (dark gray boxes), the mode rotates with a speed inversely proportional to the OAM content (as indicated by the black arrows in the time-instantaneous field distribution , while for half-integer OAM (light gray boxes), the mode resembles the two nearest integer OAM modes at time instances separated by a quarter of the oscillation period. The phase distribution ϕ(*E*_z_) (bottom row) exhibits an adiabatic shift of phase vortices (shown as gray circles) toward the center with increasing OAM content. Scale bar: one wavelength of the SPP oscillation λ_SPP_.

Figure 2 displays snapshots of the real value of the experimentally measured SPP wave function at different times of the wave oscillation as well as the retrieved phase pattern for several integer and non-integer input phase steps ν. Here, the scale bar in each snapshot corresponds to one SPP wavelength , with ϵ being the dielectric constant of the gold substrate. For integer values (highlighted in dark gray), the rotation of the positive and negative lobes of the field (red and blue maxima, respectively) with a frequency proportional to 1/ν is clearly visible, while the wave function for non-integer values exhibits a dynamic with elements discernible from both nearest integer values. With ν close to integer values, the wave function is dominated by the respective integer eigenmode, as evidenced by the number of lobes observed in the time snapshots. As ν approaches half-integer values (highlighted in light gray), a strong beating of the two adjacent integer eigenmodes can be observed, with some time instants dominated by the higher integer eigenmode and others by the lower. The switching point between the two eigenmodes during the time evolution is given by the angular choice of the phase discontinuity at the excitation boundary, which in the case shown was oriented along the *x*-direction.

As can be seen from the steady-state-like phase distributions (bottom row in Figure 2), this intricate dynamic behavior is linked to an *n*-fold split of the central phase dislocation when the excitation step ν deviates from integer values *n*, with the subsequent off-axis shift of the resulting charge +1 vortices, similar to the patterns observed in paraxial propagating light fields.^31,36–38^ The fractional nature of the plasmonic OAM mode can thus be traced to the intricate network of plasmonic vortices (represented by light and dark gray circles in Figure 2 for positive and negative charges, respectively) that expands and contracts around the central axis while at the same time exhibiting a net charge transport from the first ring of zero amplitude to the center, with vortices of charge *n* at the central axis for integer excitation steps.

### Decomposition into 2D-OAM Eigenfunctions

**Figure 3:**
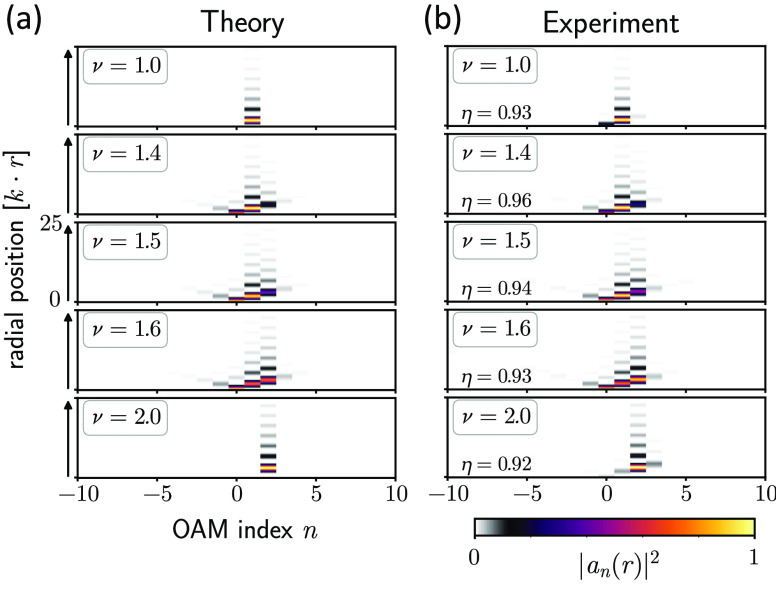
Decomposition of the plasmonic fractional OAM wave function into 2D plasmonic OAM eigenstates χ_*n*_ and their given radial dependence for different excitation phase steps ν. (a) Numerically calculated expansion coefficients *a*_*n*_(r) of the first 10 OAM eigenstates for excitation phase steps between ν = 1.0 and 2.0 (see Visualization **7**). (b) Experimentally retrieved expansion coefficients *a*_*n*_(*r*) for the same parameters as those in (a) (see Visualization **8**). The overlap integral between the experimentally determined and numerically calculated wave functions η = ⟨*A*_ν,theo_|*A*_ν,exp_⟩ is given for each phase step.

In order to better understand the evolution dynamics of fractional vortex modes and analyze their plasmonic OAM content, we decompose the experimentally obtained SPP wave field into scalar cylindrical eigenfunctions χ_*n*_ around the geometric origin, with
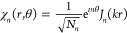
1the *n*-th eigenmode, where *k* is the SPP wavenumber, (*r*,θ) the cylindrical coordinate system, *J*_*n*_ the *n*-th order Bessel function of the first kind, and *N*_*n*_ a normalization constant determined by integrating over the full area of the eigenmode. The phase factor e^*ın*θ^ is responsible for the integer value of the OAM content of the individual eigenmodes. Decomposing the experimentally determined SPP field *E*_*z*,ν_ in terms of angular harmonics leads to radially dependent expansion coefficients *a*_*n*,ν_(*r*) = *∫e*^–*ın*θ^*E*_*z*,ν_(*r*,θ)dθ. In principle, these coefficients should have the radial dependence of the nth-order Bessel function in eq 1. In Figure 3, we visualize this decomposition for several excitation phase steps ν of the plasmonic vortices for numerically calculated (Figure 3a) as well as experimentally retrieved distributions (Figure 3b). The experimentally retrieved mode content for integer phase steps is almost exclusively given by its corresponding OAM eigenmode with a single coefficient *a*_*n*,ν_(*r*) representing the Bessel function *J*_*n*_(*kr*), as we would expect (see the top and bottom row of Figure 3b). However, a non-integer phase step results in an SPP wave consisting of many integer OAM harmonics, as observed by a fanning-out of the expansion coefficient amplitudes, highlighting the difference in spatial extent for the eigenmodes with varying OAM content (with the first zero of the Bessel functions in eq 1 shifting away from *r* = 0 with order *n*) as well as the influence of several mode orders.^21,37^ For half-integer values, this results in an equal contribution of the nearest integer eigenmodes with the radial difference between the modes responsible for the observed beating in the time evolution of the plasmonic wave field in Figure 2.

To confirm that the fractional plasmonic vortex excitations are indeed caused by a coherent linear combination of integer plasmonic OAM eigenstates, we analytically determine the 2D wave function *A*_ν_ up to an arbitrary global phase by integrating over all interfering SPP waves from the excitation boundary. Using a Fourier decomposition of the non-integer values of ν,^28^ this integral over the boundary waves can be written as a sum of the integer OAM eigenfunctions via
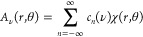
2where the coefficient *c*_*n*_ representing the probability for the plasmon to be in the OAM eigenstate *n* is
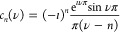


Comparing the resulting analytical mode overlap shown in Figure 3a with the experimentally retrieved distribution, we clearly see that the fanlike strength of the expansion coefficient is driven by the coherent superposition of the eigenmodes, with half-integer SPP wave functions exhibiting significant influence from more than 8 different integer OAM orders. In all examined cases, the overlap integral η between the experimentally retrieved and numerically calculated distributions is higher than 0.9.

### Mode Condensation in Ultrafast OAM Buildup

With the steady-state dynamics of the fractional plasmonic OAM field determined by the set of underlying integer eigenmodes, the intriguing question remaining is how is this fractional OAM state reached during the time-dependent buildup of the SPP wave function.

**Figure 4:**
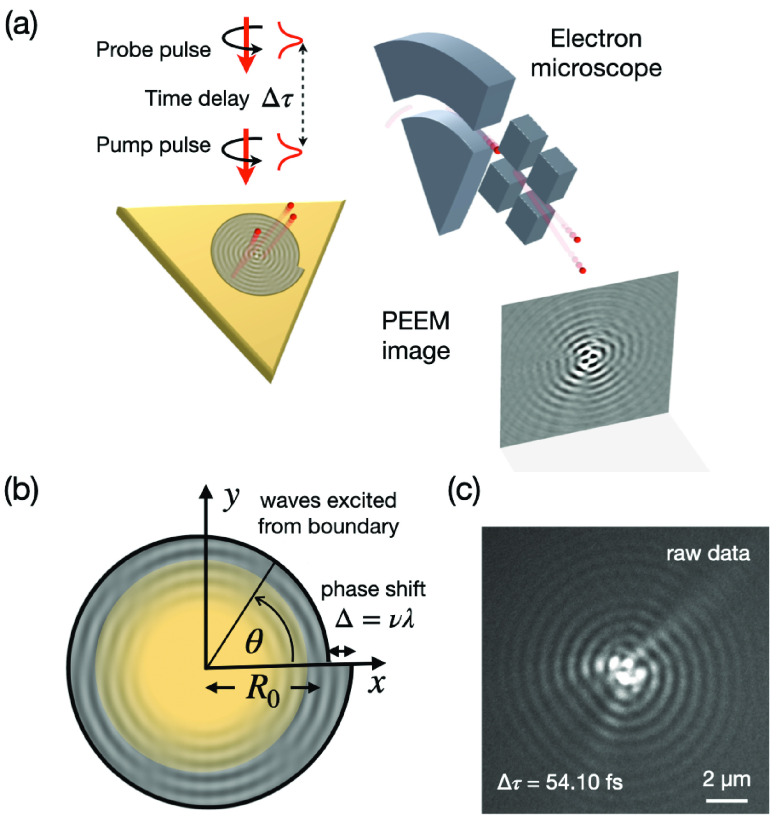
Experimental excitation and detection of the time-resolved evolution of plasmonic fractional OAM modes. (a) Sketch of the experimental PEEM setup, with pump and probe pulse both circularly polarized and extending over the whole structure. (b) Parametrization of the Archimedean spiral used to generate ultrashort plasmonic fractional OAM pulses using an initial radius *R*_0_ and phase shift Δ = νλ, as well as (c) the resulting raw intensity data for an excitation phase step of ν = 1.5 at an experimental time delay of Δτ = 54.1 fs (see also Visualization 9 for the full time evolution).

Here, we employ polarization-sensitive 2PPE–PEEM^11^ to study the local time dynamics of these plasmonic vortex modes with fractional OAM. This method combines the sub-femtosecond (sub-fs) phase resolution provided by ultrashort optical pulses with the spatial resolving power of state-of-the-art electron microscopy (see ref (28) and the Supporting Information for details about the interferometrically achieved temporal resolution), exploiting the inherently local field information of photon-mediated electron emission from conducting surfaces. We excite SPP waves via a circularly polarized 15 fs laser pulse with a central wavelength of 800 nm, illuminating the whole area of an Archimedean spiral boundary groove structured into a monocrystalline gold platelet (see Figure 4a). The excited surface waves of the conduction electrons interfere while converging toward the geometric center of the structure. They are probed after a time delay of Δτ by a second laser pulse with the same polarization state, resulting in the liberation of photoelectrons by two-photon absorption processes that subsequently form the PEEM image. Changing Δτ between the two pulses gives access to the time evolution of the excited electron distribution and, thus, the time evolution of the SPP wave function.

In our PEEM scheme, the excited SPP wave field is projected onto the probe pulse with the same polarization as the pump pulse, thus only probing the geometric structure of the interfering SPP wave function (see ref (28) and the Supporting Information). This means that the spin to OAM conversion at the groove excitation caused by the helicity of the circularly polarized pump beam, which for NSOM detection would be responsible for an additional angular-dependent phase shift, plays no role in our measured SPP wave function. Thus, we can study the purely geometric eigenfunctions of the given spiral geometry. Imprinting an angular-dependent geometric phase delay Δϕ onto the SPP waves excited at the boundary groove via the Archimedean spiral radius *r*(θ) = *R*_0_ + νλθ/2π (see Figure 4b) creates a converging SPP field with a fractional excitation phase step ν (see Figure 4c for one snapshot of a raw 2PPE–PEEM image from such an SPP distribution). The amplitude and phase information of the SPP wave function contained in the interferometric pump–probe time series can subsequently be retrieved by temporal Fourier filtering, selecting only the term with frequency components around the plasmon angular frequency ω.^28^ This allows us to visualize the complex spatial pattern of the scalar plasmon wave function *A*_ν_(*r*, θ) ∝ *E*_*z*,ν_(*r*, θ) at each time delay Δτ of the plasmonic fractional OAM pulse.

**Figure 5:**
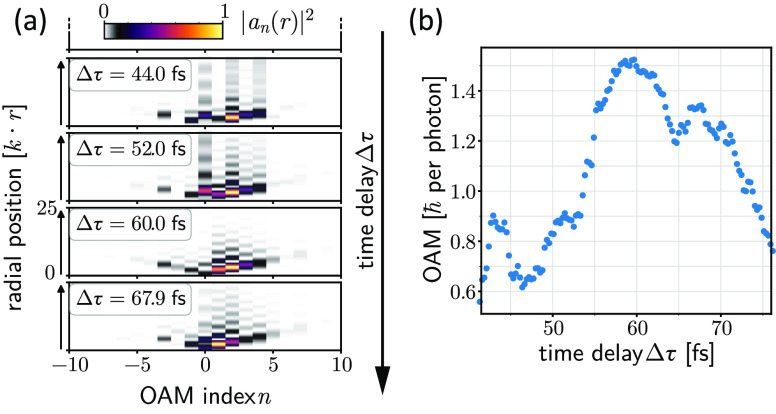
Time-resolved evolution of the plasmonic fractional OAM during the ultrafast buildup and decay for ν = 1.5. (a) Four exemplary snapshots of the eigenstate expansion coefficients *a*_*n*_(*r*) at different time delays Δτ showing the condensation of the OAM spectrum to the two dominant nearest-integer eigenstates at the point of maximum OAM (Δτ = 60 fs). (b) Evolution of the total OAM of the experimentally extracted wave function for varying time delay Δτ around the point of maximum OAM, corresponding to the steady-state case shown in Figure 3.

Due to the temporally short SPP pulses, we are able to differentiate between three distinct states of the plasmonic fractional OAM evolution: a creation phase, where the excited SPP waves travel toward the geometric origin of the Archimedean spiral; a rotation phase, where the intricate time dynamics of the created fractional vortex structure can be observed; and a decay phase.^30^ For a selected excitation phase step of ν = 1.5, we show the time-resolved evolution of the expansion coefficients during the buildup, steady state, and decay of the plasmonic fractional OAM vortex in Figure 5a. The eigenmode contribution can be observed to condense on ultrashort time scales as the steady state is reached, which is most significantly seen in the maximization of the amplitude of the two nearest integer eigenmodes. By integrating over all the contributing eigenmodes and extracting the total OAM content for each time delay Δτ of the experimentally extracted wave functions, we can follow the ultrafast buildup and decay of the OAM content with a temporal width of the OAM content on the order of the temporal width of the excitation pulses (see Figure 5b). This mode condensation and OAM content evolution are in stark contrast to the expected constant OAM content for an ideal fractional OAM state with infinite spatial extent and are to first order caused by the unavoidable background noise of the experimental realization competing with the radial decay of the evolving fractional OAM pulse (see the Supporting Information for a numerical comparison between different noise levels).

### OAM Evolution for Varying Input Phase Ramp

So far, we investigated the temporal dynamics of fractional OAM plasmons for specific values of the input phase step ν as well as their comprised off-axis phase vortices, given by a superposition of integer eigenmodes, in agreement with scalar paraxial theory. To connect the observed vortex dynamics with the interplay of intrinsic and extrinsic OAM contributions^36,39^ to the plasmonic fractional OAM, we adiabatically tune the excitation phase step ν, spanning several integer values. In addition to the experimentally retrieved values from aperture-based near-field microscopy for steps of Δν = 0.1, we perform 2PPE–PEEM measurements for selected values of ν with both left-handed and right-handed circularly polarized excitation and probe pulses to verify the handedness-independent extraction of plasmonic fractional OAM in the latter scheme.

Despite the reduced dimensionality of the plasmonic fractional OAM system, with the propagation direction of the SPP field perpendicular to the resulting OAM vector, the total angular momentum of the field can be calculated in analogy with the scalar paraxial case via its azimuthal gradient, given in operator notation as
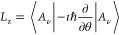
3

**Figure 6:**
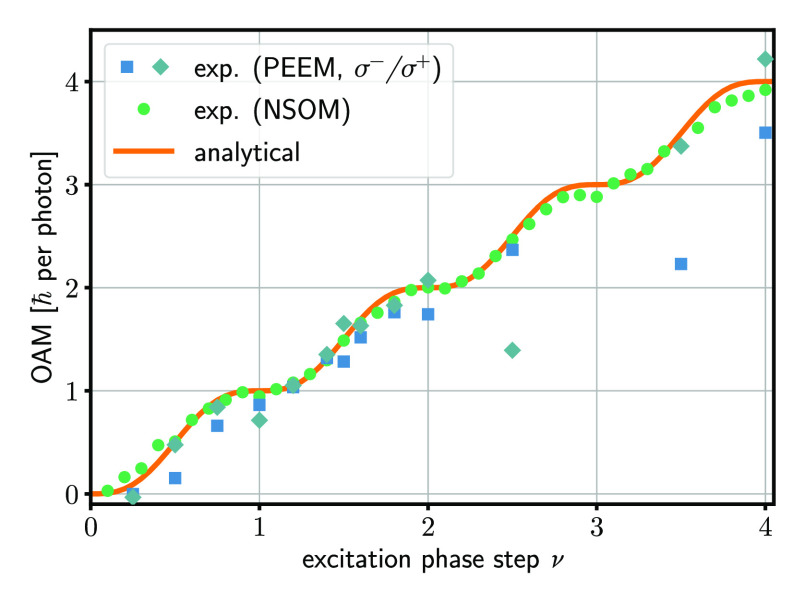
OAM evolution of plasmonic fractional OAM modes for varying phase discontinuities ν at the excitation boundary, gathered from steady-state NSOM as well as time-dynamic 2PPE–PEEM measurements. The orange line shows the theoretically expected adiabatic increase in the OAM content, with the excitation step and the OAM value matching only at points of integer and half-integer OAM. The PEEM experiments were conducted for both left- and right-handed circularly polarized excitation pulses.

For the experimentally determined wave functions of plasmonic vortex modes with phase steps ν between 0 and 4 from both steady-state near-field measurements as well as 2PPE–PEEM, the resulting fractional plasmonic OAM is plotted in Figure 6. Note that most data points of the NSOM and 2PPE–PEEM measurements coincide. The outliers in the PEEM data for ν = 2.5 and 3.5 are due to technical difficulties, mostly the presence of plasmoemission.^40^ A distinct plateau-like distribution around integer phase steps can be discerned, with the steepest increase of the OAM content at half-integer values. This is in excellent agreement with the analytical prediction of the OAM content of *L*_z_ = ℏ(ν – sin(2πν)/2π),^36^ calculated via summation over all eigenmodes weighted by the expansion coefficients *c*_*n*_(ν), with the angular momentum of the *n*-th eigenmode given by *L*_z,n_ = *ℏn*. The observed sinusoidal modulation on top of the linear OAM increase was shown for the scalar paraxial case to be given by the changing extrinsic OAM contribution due to a shift in the center-of-mass position of the given fractional OAM mode.^38^ By tracking the weighted origin of our experimentally obtained wave functions during the adiabatic tuning of the input phase step, we corroborate this interpretation for the present 2D plasmonic fractional OAM modes, confirming the physical origin of the complex total OAM as the intricate interplay between extrinsic and intrinsic plasmonic OAM.

## Conclusions

In conclusion, we experimentally visualized the complex time dynamics of 2D vortex systems in SPPs exhibiting fractional OAM, discerning their temporal creation and decay structure via 2PPE–PEEM. By accessing the full plasmon wave function, we highlight the dominant influence of the nearest integer OAM eigenstates on the fractional vortex structure while simultaneously illustrating the contributions of more than 20 eigenmode orders to the resulting SPP field. In addition, we corroborate the influence of the intrinsic and extrinsic OAM in the evolution of the wave field’s fractional nature via near-field microscopy. We envision that the presented temporal evolution of fractional plasmonic vortex modes might find application in the ultrafast addressing of on-chip quantum superposition states as well as novel tailored temporal light–matter states.

## Methods

### Sample Fabrication

The sample for near-field measurements was fabricated on a microscope cover glass by first coating a layer of 200 nm of gold via thermal evaporation. Focused ion beam milling in a FEI Helios G4 CX was performed to structure a 420 nm wide circular excitation slit of radius 75 μm into the gold surface, resulting in a circular plasmonic arena used for all shown NSOM results.

The samples for the 2PPE–PEEM measurements were single-crystalline gold flakes of 80–100 μm lateral dimension, grown via a wet chemical method^41^ on a doped silicon substrate. The sample is then placed into a Raith ionLine plus system, where spiral grooves with fractional gaps are milled using a focused Au2+ ion beam. Here, the initial radius of the Archimedean spiral was chosen as *R*_0_ = 17.5 μm.

### Near-Field Optical Measurements

Near-field measurements were performed in a home-built phase- and polarization-resolving near-field optical microscope. The excitation light from a SANTEC TSL-710 laser at λ = 1550 nm was split into a signal and reference branch for interferometric phase detection, with the signal light being spatially tailored in its polarization and phase via a SLM (Meadowlark Optics P1920). The aperture-based near-field probe consists of a tapered single-mode optical fiber coated with 180 nm aluminum and an apex opening of 200 nm. The probe-to-sample distance is controlled via shear-force feedback for scans 20 nm above the surface of the sample. The light collected through the fiber-based probe is combined with a reference beam in an optical fiber network, with the reference light frequency-shifted by 40 kHz to allow for heterodyne detection of the amplitude and phase of the collected signal. Polarization resolution is achieved by splitting the combined light beam into two orthogonal polarization components and separately detecting the amplitude and phase of the *x*- and *y*-components of the collected light field.

### 2PPE–PEEM Measurements

The time-resolved two-photon photoemission microscopy system at the University of Duisburg-Essen combines a commercial low-energy electron microscope (ELMITEC LEEM) with a femtosecond laser (FEMTOLASERS). The ultrahigh-vacuum microscope enables a spatial resolution of 11 nm. Ultrashort (12–15 fs) laser pulses are created from a Ti:sapphire oscillator with a pulse rate of 80 MHz and a central wavelength of 800 nm. Each pulse is split into two and then mutually time-delayed in a phase-stabilized Mach–Zehnder interferometer, providing a stability of better than 0.1 fs over a time of several hours. Details of the origin of the 2PPE–PEEM contrast are discussed in the study of Kahl et al.^42^

## Supporting Information Available

Generating plasmonic fractional OAM modes with arbitrary OAM content and extracting their in-plane field distribution via near-field optical microscopy, retrieving the steady-state dynamics of the full SPP wave function, extraction of the time-instantaneous information on the PEEM signal, numerical evaluation of the influence of noise on the time evolution of the fractional OAM content, visualizations of the extracted time-dependent fractional OAM fields, decomposition into OAM eigenfunctions, and example of the raw time dynamics measured via 2PPE–PEEM (PDF)
Time evolution movies of one full optical cycle, Bessel decomposition videos, and time-resolved raw data (ZIP)

## Notes

Published as part of *ACS Photonics*virtual special issue “Frontiers and Applications of Plasmonics and Nanophotonics”.
