# Supplementary material for: Ultrafast Time Dynamics of Plasmonic Fractional Orbital Angular Momentum
Source: ACS Photonics. 2023 Nov 14;10(12):4252–8. doi: 10.1021/acsphotonics.3c01036 (PMC10740006; doi:10.1021/acsphotonics.3c01036)
Supplement: Supplementary file 1 — ph3c01036_si_001.pdf [file ph3c01036_si_001.pdf]

# Supporting Information for Ultrafast time-dynamics of plasmonic fractional orbital angular momentum

**Thomas Bauer**[1,†,*], **Timothy J. Davis**[2,3,4], **Bettina Frank**[3], **Pascal Dreher**[4], **David Janoschka**[4], **Tim C. Meiler**[3], **Frank-J. Meyer zu Heringdorf**[4,*], **L. Kuipers**[1,*], **and Harald Giessen**[3,*]

[1] *Kavli Institute of Nanoscience Delft, Delft University of Technology, Delft 2628 CJ, The Netherlands*
[2] *School of Physics, University of Melbourne, Parkville, Victoria 3010, Australia*
[3] *4-th Physics Institute and Research Center SCoPE, University of Stuttgart, 70569 Stuttgart, Germany*
[4] *Faculty of Physics and Center for Nanointegration, Duisburg-Essen (CENIDE), University of Duisburg-Essen, 47048 Duisburg, Germany*
[†] *Current address: Van der Waals–Zeeman Institute, Institute of Physics, University of Amsterdam, Amsterdam 1098 XH, The Netherlands*
[*] *t.a.bauer@uva.nl, meyerzh@uni-due.de, l.kuipers@tudelft.nl, h.giessen@pi4.uni-stuttgart.de*

Number of pages: 5
Number of figures: 3

## 1. EXTRACTING IN-PLANE FIELD DISTRIBUTION OF PLASMONIC FRACTIONAL OAM STATES VIA NEAR-FIELD MICROSCOPY

To adiabatically change the OAM content of the SPP wave field, we utilize a spatially tailored vector beam impinging on a circular boundary slit structured into a gold-air interface as excitation source. Instead of 2PPE-PEEM as measurement technique, we here employ polarization- and phase-resolved near-field microscopy [1] for a continuous wave excitation light source to extract the full information of the steady-state SPP wave field. By tailoring the polarization, phase and amplitude of the field distribution at the SPP excitation slit instead of the geometry of the slit, the fractional phase step is directly encoded in the SPP field's phase distribution, allowing for a continuous variation of the phase step on one fabricated structure.

The schematic of the home-build near-field microscope is repeated from the main manuscript in Fig. S1(a) for clarity. We employ a spatial light modulator (SLM; Meadowlark Optics P1920-1100-1550) in double-pass configuration [2] to transform a Gaussian light beam at telecom wavelengths ($\lambda = 1550\,\text{nm}$; light source: Santec TSL-710) into a radially polarized beam while at the same time imprinting an azimuthal phase ramp of $2\pi\nu$ onto the doughnut shaped beam. The radial polarization is here used to allow for maximum coupling to TM-polarized SPP waves at each point of the circular excitation slit. This tailored light field is subsequently imaged from the back side onto a 200 nm thick gold film on a glass substrate containing a circular slit of radius $r = 75\,\mu\text{m}$ and a width of $w = 420\,\text{nm}$, fabricated via focused ion beam milling. A fiber-based optical near-field probe coated with 180 nm aluminium and an aperture of 200 nm at its apex (see inset in Fig. S1(a)) is raster scanned over the surface of the gold film, while its distance to the surface is controlled via shear-force feedback to ca. 20 nm. Combining the light collected through the probe with a reference wave from the same laser source, frequency shifted by 40 kHz via an acousto-optic modulator, allows us to extract amplitude and phase information via a heterodyne detection scheme. We separately detect the two orthogonal in-plane polarization components of the light field via a polarizing beam splitter after the merging of signal and reference wave, resulting in the measurement of the full vectorial in-plane field information at each scanning point of the near-field probe.

One exemplary result of such a scan over the central area of an SPP wave function with imprinted phase step of $\nu = 2.5$ is shown in figure S1(b) and (c) for the detected field amplitude of the $x$- and $y$-polarized vector component, respectively. The insets depict the relative phase

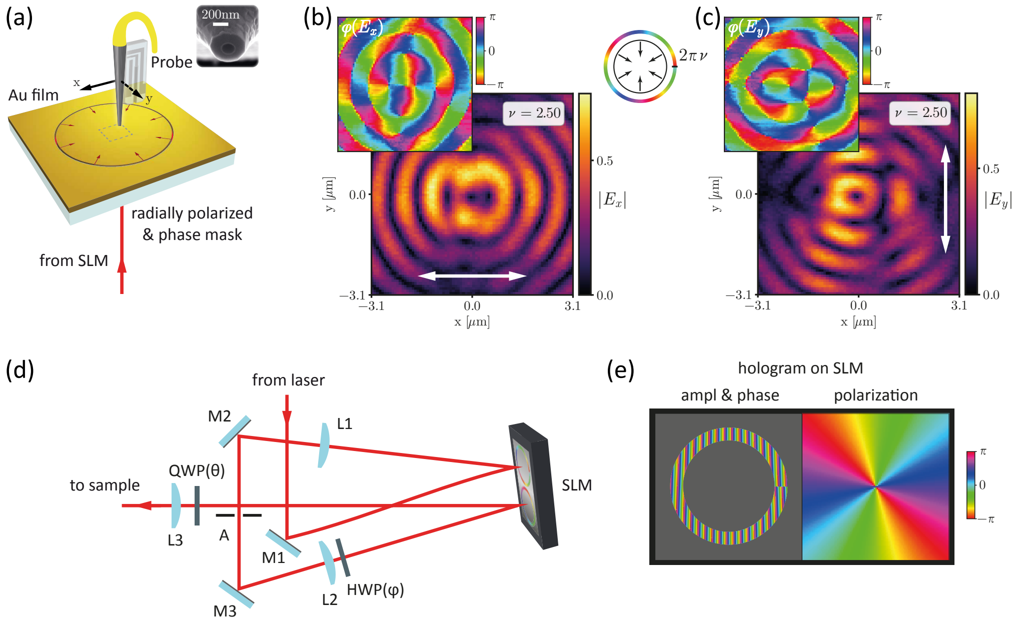


**Fig. S1.** (a) Sketch of the near-field microscope, with an aperture-based probe raster scanned over the surface of a gold film deposited on a glass substrate. SPPs are excited via a circular slit from below by a spatially tailored radially polarized light beam with imprinted phase dislocation $\nu$. Inset: SEM image of the apex of the near-field probe. (b) and (c) show the experimentally collected near-field amplitude and phase distribution of the $x$-polarized and $y$-polarized field component for an excitation phase step of $\nu = 2.5$, respectively. (d) Schematics of the double-pass configuration of the SLM to generate a radially polarized field distribution with additional azimuthal phase ramp. M1-M3: mirrors; L1, L2: lenses used in a 4f configuration to image one side of the SLM onto the other; A: aperture stop, selecting the first order of the blazed amplitude and phase hologram; HWP/QWP: half-wave and quarter-wave plate set at an angle of $\varphi$ and $\theta$, respectively. L3 is used together with another relay lens to image the final field distribution onto the backside of the sample. (e) Exemplary phase configuration of a hologram displayed on the SLM to generate an excitation field with a phase step of $\nu = 2.5$, with the left half of the hologram showing the blazed grating to modulate the amplitude and phase of the beam, while the right side imprints the desired radial polarization pattern.

distribution of each component, highlighting the intricate spatial pattern of the plasmon wave field.

Details of the optical setup to generate the radial polarization state with arbitrary additionally imprinted phase front are shown in figure S1(d). This setup follows the configuration discussed in [2], with the double pass on the SLM allowing to avoid an interferometric combination of light modulated in two orthogonal polarization states in favour of a common path for the whole field. This enhances the phase stabilization of the setup, with one half of the SLM used to modulate the amplitude and phase of the local fields while the other half imprinting the desired polarization state that can be selected from an arbitrary cut through the Poincare sphere via two waveplates [2]. For the generation of a radially polarized distribution, the first waveplate is a half-wave plate set under an angle $\varphi = 3\pi/8$ with respect to the x-axis, while the second waveplate is a quarter-wave plate set under an angle $\theta = -\pi/4$ with respect to the x-axis. The aperture A filters the first diffraction order of the blazed grating displayed in the amplitude and phase hologram (see Fig. S1(e)), while the lenses realize a 4f configuration to image the amplitude and phase hologram onto the polarization hologram on the other half of the SLM. Any desired phase step $\nu$ can be imprinted on top of the blazed amplitude and phase hologram.

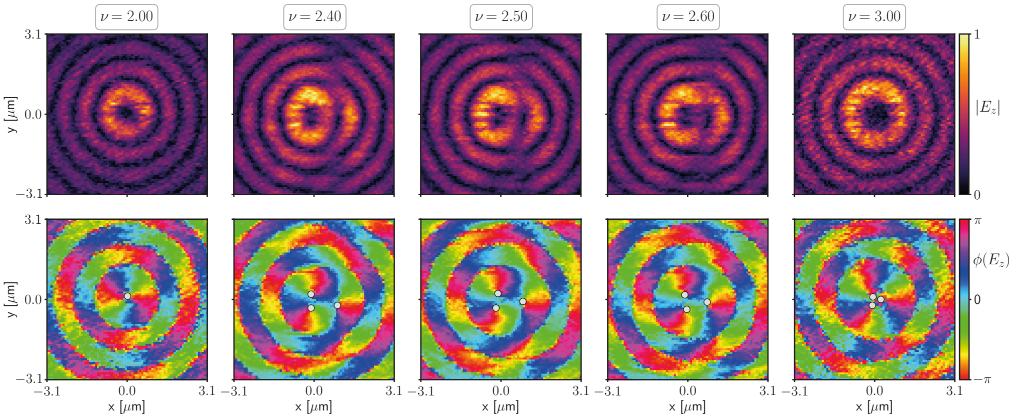


**Fig. S2.** Experimentally retrieved amplitude and phase distribution of the $E_z$ component of the plasmonic wave function for several excitation phase steps of $\nu$ between the integer values of 2 and 3. The phase dislocation was aligned in the $+x$-direction. The light gray circles in the phase distributions correspond to plasmonic vortices of charge $+1$, highlighting the addition of one vortex from the first ring of zero amplitude to the geometric center of the distribution along the $-x$-direction as the excitation phase step $nu$ is increased.

## 2. RETRIEVING STEADY-STATE DYNAMICS OF FULL SPP WAVE FUNCTION FROM IN-PLANE FIELD COMPONENTS

From the phase-resolved detection of $E_x$ and $E_y$, we unambiguously reconstruct $E_z$ and thus the full wave function due to the SPPs consisting of purely TM waves, which we elaborate on in the following. Assuming a general monochromatic TM mode ($H_z = 0$) bound to an interface between gold and air in the $z$-direction, with global $z$-dependence of $\exp(\imath k_z z)$ and a temporal evolution of the wave as $\exp(-\imath\omega t)$, Maxwell's equations in free space above the interface allow us to write for the in-plane electric and magnetic field components as

$$E_x = \frac{\imath}{\epsilon_0\omega}\left(\partial_y H_z - \partial_z H_y\right) = \frac{\imath k_z}{\epsilon_0\omega} H_y, \tag{S1}$$

$$E_y = \frac{\imath}{\epsilon_0\omega}\left(\partial_z H_x - \partial_x H_z\right) = -\frac{\imath k_z}{\epsilon_0\omega} H_x, \tag{S2}$$

with $k_z = \frac{2\pi}{\lambda}\sqrt{1-\frac{\epsilon}{1+\epsilon}}$ for a free-space wavelength $\lambda$ and $\epsilon$ the dielectric constant of the gold substrate. Here, $k_z$ is derived from the SPP dispersion relation $k_{\text{SPP}} = \frac{2\pi}{\lambda_{\text{SPP}}} = \frac{2\pi}{\lambda}\sqrt{\frac{\epsilon}{1+\epsilon}}$. This means that the in-plane components of the electric and magnetic field are uniquely linked for any SPP wave field. Furthermore, due to the divergence-free electric field in free space, the $E_z$ component above the interface is given by

$$E_z = \frac{\imath}{k_z}\left(\partial_x E_x + \partial_y E_y\right). \tag{S3}$$

The thus extracted amplitude and phase distribution of the SPP wave function (which is equivalent to $E_z$) is shown for several excitation phase discontinuities $\nu$ between 2 and 3 in figure S2. The phase distributions highlight the evolution from a central phase vortex of charge 2 to a vortex of charge 3 by the continuous shift of a further phase vortex from the surrounding towards the center of the distribution along the $-x$-direction.

We can determine the total angular momentum of each of these wave functions by applying equation (3) of the main manuscript for $A_\nu = E_{z,\nu}$, or equivalently utilizing the in-plane current flow $\mathbf{p}_\perp$ of the scalar wave [3] and evaluating the classical definition of angular momentum as

$$\mathbf{p}_\perp = |E_z|^2 \nabla_\perp \arg\left(E_z\right), \tag{S4}$$

$$J_z = \frac{\int \mathbf{r}\times\mathbf{p}_\perp \mathrm{d}x\mathrm{d}y}{\int |E_z|^2 \mathrm{d}x\mathrm{d}y}. \tag{S5}$$

The resulting OAM values retrieved from the distributions in figure S2 as well as distributions for other excitation phase steps $\nu$ via this approach are shown in figure 4 of the main manuscript.

## 3. EXTRACTING TIME-INSTANTANEOUS PHASE INFORMATION VIA INTERFEROMETRIC PUMP-PROBE PEEM

In order to understand the ability to extract the purely geometric wave function from 2PPE-PEEM measurements with sub-femtosecond resolution of its phase evolution, we summarize here the results of reference [4]. Considering for simplicity one edge generating a surface plasmon propagating in the direction $\hat{n}$ with an amplitude $E_p$ that is small compared to the incident light amplitude $E_l$ exciting the plasmon, and the two light pulses (pump and probe) in the same polarization state $\hat{e}$, the electron emission can be shown to be proportional to

$$I^2 = |\mathbf{E}_l + \mathbf{E}_p|^4 \sim |\mathbf{E}_l|^2 \left( |\mathbf{E}_l|^2 + 4\Re(\mathbf{E}_l^* \cdot \mathbf{E}_p) \right) \tag{S6}$$

with terms of higher order in $\mathbf{E}_p$ ignored. Assuming that the delay $\Delta\tau$ between pump and probe pulse is long enough that the two light pulses don't overlap, a time integration over the resulting PEEM signal only needs to retain the spatially constant background of the individual pulses as well as the interference term between the plasmon excited by the pump pulse and the time-delayed probe pulse. This results in the PEEM signal $P$ (see also eqn. (14) in Ref. [4]):

$$P(\Delta\tau, \mathbf{r}) \sim C(\mathbf{r}) - A|\hat{e} \cdot \hat{n}|^2 E_1 E_2^3 \sin(k|\mathbf{s}| - \omega\Delta\tau) \mathrm{e}^{-3(k|\mathbf{s}| - \omega\Delta\tau)^2/\omega^2\tau_0^2} \tag{S7}$$

with all time-independent signal contributions given by $C(\mathbf{r})$, and the interference between the plasmon excited by the first light pulse with amplitude $E_1$ and the second light pulse with amplitude $E_2$ delayed by a time $\Delta\tau$ leading to a sinusoidal variation of the PEEM signal with a period equal to the plasmon wavelength, appearing like a traveling wave that depends on the set delay time. Here, $\mathbf{s} = \mathbf{r} - \mathbf{r_0}$ is the vector from excitation to detection point, $\omega$ is the central frequency of the light pulses with pulse length $\tau_0$, and $k$ is the wave vector of the propagating plasmon, with $A$ a time-independent amplitude coefficient of the interference pattern.

From the sinusoidal variation depending on $\Delta\tau$, it can be seen that by sweeping the relative time delay between pump and probe pulse, the full time dependence of the plasmon wave function including its instantaneous phase can be extracted. In addition, the dependence of the electron yield on $|\hat{e} \cdot \hat{n}|^2$ means that for a circularly polarized pump and probe pulse with same handedness $\hat{e} = (\hat{x} \pm \imath\hat{y})/\sqrt{2}$, the light's helicity does not play a role in the generated PEEM signal, irrespective on the orientation of the excitation boundary. Thus, the described approach probes the purely geometric plasmon wave function, with the spin to orbital angular momentum conversion at the plasmon excitation boundary canceled by the helicity of the probe pulse.

## 4. NUMERICAL EVALUATION OF THE TIME-DEPENDENT BUILD-UP OF THE SPP WAVE FUNCTION

While the experimentally retrieved time dynamics of the fractional OAM wave function shows a clear mode condensation and ultrafast OAM build-up (see main text, Fig. 5), this behaviour is quite counter-intuitive. Considering a mode spanning the full 2D plane and a perfect noise-free system, one would intuitively expect a fixed OAM content independent of the time evolution of the wave function, with the eigenmode decomposition after creation of the wave function staying constant. However, any experimental realization has to incorporate the finite extent of the excited SPP wave as well as background noise from both, fundamental fluctuations as well as scattering sites in the employed material.

Assuming for simplicity a scalar Gaussian wave packet with amplitude $A_0$, central wavelength $\lambda = 800\,\mathrm{nm}$ and pulse width $\tau_0 = 10\,\mathrm{fs}$ being launched from a boundary at radius $R_0 = 17.5\,\mu\mathrm{m}$ and imprinted with an excitation phase with phase step $\nu = 1.5$, a noise-free system would indeed result in a near-constant fractional OAM value as the pulse evolves through its creation, rotation and decay phase (see Fig. S3(a)). However, adding a normal distributed amplitude noise to the numerically calculated wave function, it can be seen that even an amount of $\sigma_A = 0.1A_0$ drastically changes the fractional OAM content as the pulse evolves, with the resulting OAM build-up and decay during the creation and decay phase of the SPP pulse governed by the competition between the amplitude of the background fluctuations and the $1/r$ decay of the radially inward/outward moving pulse.

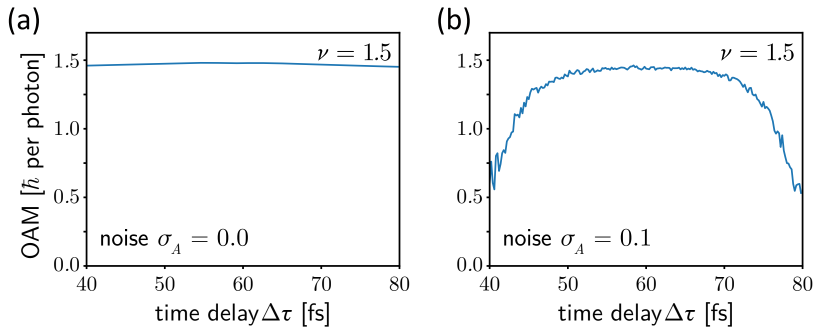


**Fig. S3.** Numerically calculated time evolution of the OAM content of a scalar 2D wave packet $A(r,t)$ for different amounts of background noise $\sigma_A$. The Gaussian wave packet with amplitude $A_0$, temporal width $\tau_0 = 10\,\text{fs}$ and central wavelength $\lambda = 800\,\text{nm}$ is excited on a ring of radius $R_0 = 17.5\,\mu\text{m}$ and is imprinted with an azimuthally varying phase with phase step $\nu = 1.5$. (a) A perfect noise-free system is assumed. (b) Amplitude noise with a normal distribution width $\sigma_A = 0.1A_0$ is added to the simulation domain.

## REFERENCES


1. M. Burresi, R. Engelen, A. Opheij, D. van Oosten, D. Mori, T. Baba, and L. Kuipers, "Observation of Polarization Singularities at the Nanoscale," Phys. Rev. Lett. **102**, 033902 (2009).
2. E. Otte, C. Schlickriede, C. Alpmann, and C. Denz, "Complex light fields enter a new dimension: holographic modulation of polarization in addition to amplitude and phase," in *Proceedings of SPIE,* vol. 9379 E. J. Galvez, J. Glückstad, and D. L. Andrews, eds. (2015), p. 937908.
3. M. V. Berry, "Optical currents," J. Opt. A: Pure Appl. Opt. **11**, 094001 (2009).
4. T. J. Davis, B. Frank, D. Podbiel, P. Kahl, F.-J. Meyer zu Heringdorf, and H. Giessen, "Subfemtosecond and Nanometer Plasmon Dynamics with Photoelectron Microscopy: Theory and Efficient Simulations," ACS Photonics **4**, 2461–2469 (2017).
